# SUBJECTIVE AGE AND FUTURE LONELINESS: COMPARING CAREGIVERS AND NON-CAREGIVERS ACROSS TEN YEARS

**DOI:** 10.1093/geroni/igac059.377

**Published:** 2022-12-20

**Authors:** Rachel Koffer, Hannah Giasson

**Affiliations:** Arizona State University, Phoenix, Arizona, United States; Arizona State University, Tempe, Arizona, United States

## Abstract

Experiences that make age salient can shape how old or young a person feels relative to their chronological age. Caregiving for a loved one may contribute to subjective age as well as the linkages between subjective age and well-being. We examined longitudinal associations between subjective age and future loneliness ten years later among 2,557 caregivers and non-caregivers (Baseline Mage=55) in the Midlife in the United States Study (2004-2014). Linear regression results indicated an interaction between caregiver status and subjective age (β = 0.60, 95% CI=[0.14, 1.06]), such that among non-caregivers, older subjective age predicted greater future loneliness, but among caregivers, younger subjective age predicted greater future loneliness. Associations hold after controlling for age, baseline loneliness, and caregiver status at follow-up. Future subjective aging research should examine the unique context of caregiving to further understand the meaning of subjective age and how it predicts important well-being outcomes among caregivers.

